# Role of Lopinavir/Ritonavir in the Treatment of Covid-19: A Review of Current Evidence, Guideline Recommendations, and Perspectives

**DOI:** 10.3390/jcm9072050

**Published:** 2020-06-30

**Authors:** Simone Meini, Alberto Pagotto, Benedetta Longo, Igor Vendramin, Davide Pecori, Carlo Tascini

**Affiliations:** 1 Struttura Operativa Complessa di Medicina Interna, Azienda Unità Sanitaria Locale Toscana Centro, Ospedale Santa Maria Annunziata, 50012 Firenze, Italy; simonemeini2@gmail.com; 2 Clinica di Malattie Infettive, Azienda Sanitaria Universitaria Friuli centrale, 33100 Udine, Italy; alberto.pagotto@asufc.sanita.fvg.it (A.P.); davide.pecori@asufc.sanita.fvg.it (D.P.); 3 Struttura Operativa Complessa di Medicina Interna, Azienda Unità Sanitaria Locale Toscana Nord-Ovest, Ospedale Felice Lotti, 56025 Pontedera, Italy; afataaa@gmail.com; 4 Dipartimento Cardiotoracico, Divisione di Cardiochirurgia, Azienda Sanitaria Universitaria Friuli centrale, 33100 Udine, Italy; igor.vendramin@asufc.sanita.fvg.it

**Keywords:** lopinavir, ritonavir, COVID-19, SARS, MERS, guideline, recommendation

## Abstract

A life-threatening respiratory illness (COVID-19) due to severe acute respiratory syndrome (SARS)-CoV-2 coronavirus was first described in December 2019 in Wuhan (China), rapidly evolving into a pandemic. In the first phase, when the viral replication plays a pivotal pathogenetic role, antiviral drugs could be crucial in limiting viral-induced organ damage. Unfortunately, there are no specific antivirals of proven efficacy for COVID-19, and several drugs have been repurposed to face this dramatic pandemic. In this paper we review the studies evaluating lopinavir/ritonavir association (LPV/r) use in COVID-19, and previously in SARS and Middle East respiratory syndrome (MERS). We searched PubMed to identify all relevant clinical and laboratory studies published up to 15 May 2020; the guidelines on the use of LPV/r in COVID-19 were further directly searched on the website of the main international scientific societies and agencies. Available evidence is currently scarce and of low quality. The recommendations issued for COVID-19 vary from positions clearly against the use of LPV/r to other positions that are more favorable. In our opinion, despite the controversial results of an important randomized clinical trial, and some recommendations, clinicians should not abandon the use of LPV/r for the treatment of COVID-19, possibly using this drug inside a prospective randomized trial, waiting for the results of the numerous ongoing trials evaluating its efficacy.

## 1. Introduction

Starting in December 2019 in Wuhan (Hubei province, China), a novel coronavirus (CoV), designated severe acute respiratory syndrome (SARS)-CoV-2, caused an international outbreak of a respiratory illness (COVID-19), and rapidly evolving into a pandemic. Most cases are asymptomatic or self-limiting, but the clinical spectrum of the disease extends to severe progressive pneumonia with acute respiratory distress syndrome (ARDS), which is a life-threatening condition requiring mechanical ventilation and intensive care support.

In a yet to be defined percentage of cases, after about one week, there is a sudden and unpredictable worsening of clinical conditions, properly having as a target the respiratory district. In the first phase of the infection, when the viral replication plays a pivotal pathogenetic role, the use of antiviral drugs could be of the utmost importance in inhibiting SARS-CoV-2 replication and the viral-induced organ damage; unfortunately, to date there are no antiviral therapies specific for COVID-19.

Some antivirals initially developed as treatment for human immunodeficiency virus (HIV), including lopinavir/ritonavir association (LPV/r), have been investigated in the past two decades (drug repurposing) for other coronaviruses responsible for serious diseases such as the severe acute respiratory syndrome (SARS) and the Middle East respiratory syndrome (MERS) [1,2]. In a recent meta-analysis aimed at evaluating the efficacy and safety of current options for SARS and MERS besides COVID-19, the LPV/r-based combinations showed better virological eradication and radiological improvement, with a reduced rate of ARDS as compared with other anti-coronavirus agents [3]. In the absence of proven effective treatments, LPV/r has, thus, been widely used to face the COVID-19 pandemic from the beginning [4], and is currently investigated in several clinical trials [5], such as the Solidarity trial launched on 20 March by the World Health Organization [6].

The aim of this paper is to review the available evidence on the efficacy of LPV/r in COVID-19, SARS, and MERS, and to analyze the current guideline recommendations of the main international scientific societies and agencies on the use of this antiviral drug.

## 2. Methods

A literature search was performed up to 15 May 2020 through PubMed. The following terms were searched in different combinations: COVID-19, SARS-CoV-2, SARS, MERS, coronavirus, lopinavir, and ritonavir. Variations of these terms were also searched. The selection was limited to articles written in English and published in peer-reviewed journals only. After deduplication, all authors independently screened titles and abstracts, and finally full texts, to identify all potentially relevant papers, resolving discrepancies through discussion and consultation among them. References of retrieved articles were manually searched to ensure identification of relevant studies not found in the initial literature search.

The guideline documents of the main scientific societies involved in the management of COVID-19, and of international agencies, were directly searched and retrieved through direct consultation of their websites, in addition to PubMed searching.

Finally, a search was performed on https://clinicaltrials.gov for the studies regarding the use of lopinavir/ritonavir in COVID-19, in progress or waiting to begin, or completed but not yet published.

## 3. Results

### 3.1. Literature Search

**Figure 1:**
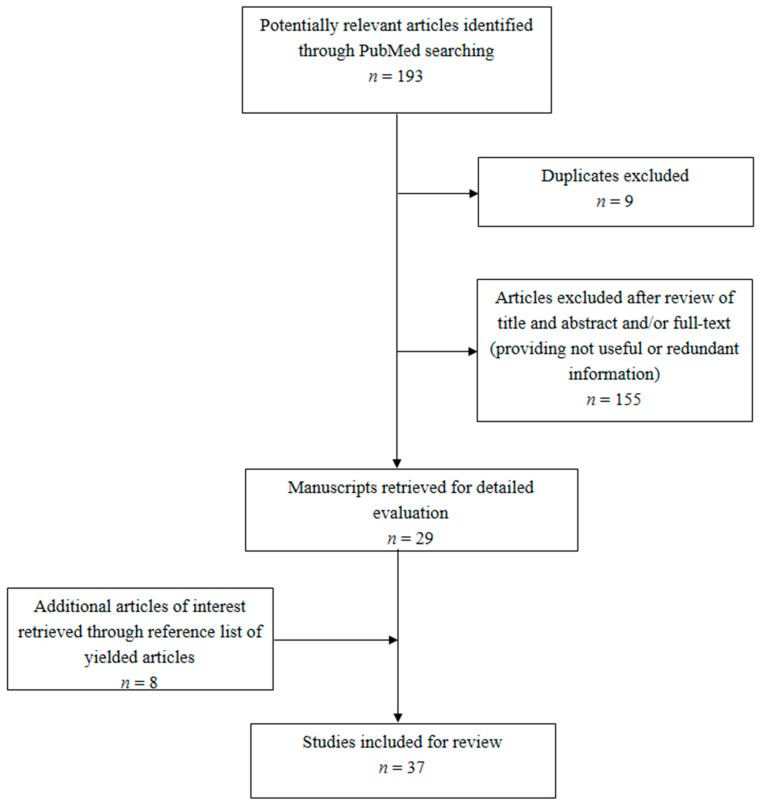
Flow chart of the literature selection process regarding clinical and laboratory studies on lopinavir/ritonavir association (LPV/r) use in COVID-19, severe acute respiratory syndrome (SARS), and Middle East respiratory syndrome (MERS).

Figure 1 shows the flow diagram of the literature selection process to identify clinical and laboratory studies regarding LPV/r use in COVID-19, SARS, and MERS. Thirty-seven studies were included for the review. Thirteen documents released by some authoritative scientific societies and international agencies involved in the management of COVID-19 were retrieved.

### 3.2. Lopinavir/Ritonavir: An Overview

Lopinavir (LPV) is an antiretroviral protease inhibitor used in combination with ritonavir (booster) in HIV infection therapy and prevention. LPV is a peptidomimetic HIV type 1 aspartate protease inhibitor that acts by binding to its catalytic site, thereby, preventing the cleavage of viral polyprotein precursors into mature, functional proteins that are necessary for viral replication. LPV is usually given in combination with low booster doses of ritonavir which improves the pharmacokinetics of LPV by slowing its hepatic metabolism through the inhibition of cytochrome P450 3A4 enzyme.

The standard recommended dosage of LPV in adults is 800 mg daily in combination with 200 mg of ritonavir, usually in two divided doses.

Some degree of serum aminotransferase elevations occurs in a high proportion of patients within one to eight weeks, usually asymptomatic and self-limiting with continuation of the medication; however, fatal cases have been reported [7]. Comprehensive additional information about LPV/r can be found at the website https://www.uptodate.com/contents/lopinavir-and-ritonavir-drug-information.

LPV/r was developed for inhibition of HIV protease, whose main difference with respect to the SARS-CoV-2 counterpart (3CL^pro^) lies within the divergent spatial structure of the HIV aspartic protease as compared with 3CL^pro^ cysteine protease. This would affect its inhibition efficiency [8].

Recently, it has been pointed out by Baldelli et al. [9] that to establish the optimal dosage of LPV/r in COVID-19 could be very relevant, although it has been completely overlooked so far: in 21 patients concomitantly treated with hydroxychloroquine, all the LPV therapeutic drug monitoring (TDM) resulted in values above the therapeutic range, and the TDM data analysis showed that the COVID-19 patients had trough LPV concentrations three-fold higher as compared with the HIV patients. Virtually all samples exceeded the threshold concentration of 7000 ng/mL, notoriously associated with scarce tolerability in HIV patients. The high concentrations both of LPV and ritonavir could be explained by the SARS-CoV-2-induced liver damage, including the altered expression of drug-metabolizing enzymes, or by the potential interaction with hydroxychloroquine. The authors wisely suggest that an investigation is warranted for a reduced dose of LPV/r (i.e., 400/100 mg daily) to improve drug tolerability in COVID-19 [9].

### 3.3. Current Evidence for LPV/r in SARS

The ability of LPV to inhibit the SARS-CoV main protease (3CL^pro^) has been evaluated in vitro [10].

No clinical trials were found addressing the efficacy of antiviral agents in the management of SARS-CoV [11].

Two observational studies reported outcomes in patients who were given LPV/r. In a retrospective matched cohort study conducted in Hong Kong [12], the addition of LPV/r as initial treatment in 44 patients was associated with a reduction of the overall death rate (2.3%) and intubation rate (0%) as compared with a matched cohort of 634 patients who received standard treatment (15.6% and 11.0% respectively, *p* < 0.05) and a lower rate of use of methylprednisolone at a lower mean dose. The subgroup of 31 patients, who received LPV/r as a rescue therapy, showed no difference in overall death rate and rates of oxygen desaturation and intubation as compared with a matched cohort of 343 patients, and received a higher mean dose of methylprednisolone. In another study [13], LPV/r was administered in addition to ribavirin to 41 patients. Adverse clinical outcomes (ARDS or death) at 21 days were significantly lower than in 111 historical controls treated with ribavirin only (2.4% vs. 28.8%, *p* < 0.001) three weeks after the onset of symptoms, and this positive effect was shown in those diagnosed both early and later in the course of the epidemic. A reduction in steroid usage and nosocomial infections and decreasing viral loads were seen in patients initially treated with LPV/r. Focusing on multivariate analysis, the lack of treatment with this antiviral was an independent predictor of adverse outcomes. LPV showed in vitro antiviral activity at 48 h at concentrations of 4 μg/mL.

However, both these studies were determined to be “inconclusive” due to the possible bias in the selection of the control group or treatment allocation [14].

### 3.4. Current Evidence for LPV/r in MERS

In an in vitro study [15], LPV inhibited MERS-CoV-induced cytopathic effect with a 50% effective concentration (EC_50_) of 8 μM, and a maximal protective effect (89% inhibition) was observed at a dose of 12 μM. These values are in the range of the LPV plasma concentrations (8 to 24 μM) observed in HIV patients with acquired immune deficiency syndrome (AIDS).

No published clinical trials were found addressing the efficacy of antiviral agents in the management of MERS-CoV [11].

From May to July 2015, the Republic of Korea experienced the largest outbreak of MERS outside the Arabian Peninsula: a retrospective observational study focused on the clinical characteristics of confirmed MERS cases [16]. An antiviral therapy was administered to 138/186 patients, within a median time of six days from the onset of illness, and LPV/r was commonly prescribed to 86.6% of patients, usually in combination with interferon and ribavirin (81.1%) or ribavirin only (5%), and as a monotherapy in 0.8% only. Since the case fatality rate observed in this study was lower than that reported in other studies of previous MERS outbreaks (20.4% vs. 36.5–65%), the authors suggested that this low rate could be attributed to the application of more aggressive treatment measures, including the antiviral agents.

In a nonhuman primate model (common marmoset) of MERS, animals treated with LPV/r and interferon-β 1b had a better outcome than the untreated, with improved clinical, radiological, and pathological findings, and lower mean viral loads in lung and extra-pulmonary tissues [17].

The MIRACLE (MERS-CoV Infection tReated with A Combination of Lopinavir/ritonavir and intErferon-β 1b, ClinicalTrials.gov ID NCT02845843) [18] is a multicenter double-blind randomized placebo-controlled trial (RCT) that is currently recruiting patients to investigate the efficacy (the primary outcome is 90-day mortality) of this combination therapy in hospitalized patients. The MIRACLE trial is the first RCT performed for MERS treatment.

The Public Health England recommendations on “Treatment of MERS-CoV” stated that “LPV may be considered for specific treatment of MERS patients (…), routinely available (…), likely to be the most accessible treatment initially (…), well-established agent with favorable toxicity profile. Gastrointestinal side effects are common but self-limiting” [19].

### 3.5. Current Evidence for LPV/r in COVID-19

Crystal structure of SARS-CoV-2 protease has been released, providing the structural basis for identification of drugs that could interact with this target. Computer-aided drug design can play an essential role to identify new active drugs. LPV displayed inhibitory activities against SARS-CoV-2 main protease [20]. The role of the nonstructural protein of coronaviruses, main protease, or 3C-like proteases (3CL^pro^), involves the proteolytic processing of the replicase polyprotein, and is crucial for viral replication and maturation; this protease shares a similar common cleavage site among coronaviruses, and the sequence alignment of SARS-CoV-2 3CL^pro^ shows a 96.1% identity as compared with that of SARS-CoV. Coronavirus 3CL^pro^ is considered to be an attractive drug target for the treatment of coronavirus infections. Both LPV and ritonavir effectively interact with the residues at the active site of SARS-CoV-2 3CL^pro^. Ritonavir shows a somewhat higher number of atomic contacts, a somewhat higher binding efficiency, and a somewhat higher number of key binding residues as compared with LPV, which corresponds with the slightly lower water accessibility at the 3CL^pro^ active site [21].

A recent study [22] showed that both remdesivir and LPV (but not ritonavir) inhibited SARS-CoV-2 replication in Vero E6 cells, with the estimated EC_50_ at 23.15 and 26.63 μM, respectively, while other compounds currently undergoing clinical trials (such as ribavirin, favipiravir, oseltamivir, or baloxavir) showed no apparent in vitro antiviral effect at concentrations under 100 μM.

In the first retrospective single-center study describing the clinical characteristics of 98 hospitalized COVID-19 patients in South Korea, 99% of the patients received LPV/r [23], as well as 82% of the critically ill patients hospitalized in Brescia, Italy [24], testifying how this antiviral drug has been widely used in clinical practice, worldwide, to face this dramatic illness.

Wu et al. [25] evaluated risk factors for ARDS in 201 COVID-19 patients, most of whom (84.6%) received different antiviral drugs, including LPV/r (14.9%). Patients who developed ARDS were less likely to be treated with antiviral therapy (difference, −14.4%, 95% CI, −26.0% to −2.9%, *p* = 0.005), and the same occurred in those who died (difference, −40.7%, 95% CI, −58.5% to −22.9%, *p* < 0.001). However, the low number of patients treated with LPV/r did not allow them to make any conclusion on the efficacy of this drug.

LPV/r has been reported to be associated with faster resolution of fever, with no evident toxicity and only low side effects (mainly nausea, vomiting, and diarrhea), and with fewer days for SARS-CoV-2 RNA detection turning negative [26]. It has been suggested that LPV/r can reduce viral loads [27], and that the discontinuation of antiviral drugs, including LPV/r, could be the reason for recovered COVID-19 patients testing positive again, with new worsening of pulmonary damage [28]. However, it has also been shown that LPV/r did not shorten the duration of SARS-CoV-2 shedding in patients with mild pneumonia [29]. It has been reported a significantly more elevated negative conversion rate of coronavirus test in seven days and 14 days, and a significant improvement of chest radiological findings in seven days, when LPV/r was administered in combination with oral arbidol [30]. In another retrospective study, LPV/r (400/100 mg twice a day for one week) was reported to be inferior to arbidol in terms of viral load reduction and duration of positive RNA testing [31].

A specific focus on LPV/r administration was done in a descriptive case series of the first 18 patients diagnosed in Singapore. Five patients requiring supplemental oxygen were treated with LPV/r, but the evidence of clinical benefit was equivocal, since defervescence and reduction of supplemental oxygen occurred in three patients within one to three days of LPV/r initiation, but it was unable to prevent the progression of the disease in the remaining two patients. In addition, 4/5 patients developed nausea, vomiting, or diarrhea, and three patients developed an abnormal liver function test. A decline in viral load in nasopharyngeal swabs also appeared to be similar between treated and untreated patients [32].

The efficacy of LPV/r has been specifically investigated in a randomized controlled open-label trial by Cao et al. [33], in which 99 patients with severe COVID-19 were randomized to receive LPV/r (400/100 mg twice a day) and 100 patients to standard of care, for 14 days. LPV/r administration was not associated with a statistically significant difference in the time to clinical improvement (the primary outcome), although in the modified intention-to-treat analysis, excluding three patients who died within 24 h and who never received the medication, the between-group difference (median, 15 days vs. 16 days) was significant, albeit modest. Moreover, the median time when the patients participated in the study was 13 days after symptom onset, when they had already progressed to a very severe condition (not excludable, being secondary to virus-induced systemic inflammatory response due to cytokine release syndrome). In a post-hoc subgroup analysis, the difference in mortality between the LPV/r group and the standard-care group was observed to be numerically greater among patients treated within 12 days after the onset of symptoms than among those treated later. In addition, in the LPV/r group as compared with the standard-care control group: the 28-day mortality was lower (19.2% vs. 25%, but the difference was not statistically significant), patients presented a shorter stay in the intensive care unit (median, six days vs. 11 days), the duration from randomization to hospital discharge was numerically shorter (median, 12 days vs. 14 days), the percentage of patients with clinical improvement at day 14 was higher (45.5% vs. 30.0%), the risk of respiratory failure or ARDS was lower (12.6% vs. 27.3%), secondary infections were lower (1.1% vs. 6.1%); moreover, in the LPV/r group as compared with the standard-care control group, a lower use of vasopressors (17.2% vs. 27.0%), renal-replacement therapy (3.0% vs. 6.0%), noninvasive (10.1% vs. 19.0%) and invasive (14.1% vs. 18.0%) mechanical ventilation was reported. The viral RNA load or duration of viral RNA detectability did not differ between the two groups. LPV/r was associated with more adverse events, in particular gastrointestinal adverse events, including anorexia (2.1%), nausea (9.5%), abdominal discomfort (4.2%), vomiting (6.3%), or diarrhea (4.2%), as well as two serious adverse episodes of acute gastritis. Increased levels of transaminases were instead less frequently reported in the LPV/r group than in the controls. This was not a blinded trial, and the small population enrolled was the main limitation of this trial; it was likely that if the RCT had been larger, some of the favorable point estimates could have reached statistical significance. Moreover, the enrolled population had severe disease (overall mortality of 22%), and this could have contributed to the poor effect associated with the treatment; this highlights the importance of an early antiviral approach when in the pathogenesis of COVID-19 the “viral replication phase” prevails over the “host immune response”. Finally, the somewhat higher throat viral loads in the LPV/r group raise the possibility that this group had more viral replication.

After the publication of this RCT, many clinicians stopped using LPV/r, with some scientific societies even recommending against its use.

Many authors suggested a different reading and alternative analyses of the results of this trial. It was highlighted by Corrao et al. [34] that even if the results of a trial have no statistical significance, signals are important if the sample size is small, and no strong conclusions can be made until a trial with the correct sample size has been performed. Carmona-Bayonas et al. [35] reanalyzed the data of Cao et al. [33] with a Bayesian Cox proportional hazards model and showed that there was a 73% posterior probability of a clinical improvement of more than 15% and a 17% probability that the effect was in a region of practical equivalence. Since this trial was underpowered, the results did not sustain the conclusion that LPV/r was ineffective. Cao et al. [36] recently admitted that on the basis of the results regarding different outcomes and alternative data analyses, LPV/r could still be a potential therapeutic agent against COVID-19, and wisely recommended that clinicians review all the data about all the outcomes in their trial before making any clinical decisions regarding treatment.

A recent multicenter, prospective, open-label, randomized, phase II trial [37] showed that 86 hospitalized patients early assigned (within a median of five days) to a 14 day triple combination of LPV/r 400/100 mg every 12 h, ribavirin 400 mg every 12 h, and three doses of 8 million international units of interferon-β 1b on alternate days, had a significantly shorter median time from start of treatment to negative nasopharyngeal swab as compared with a control group of 41 patients treated only with LPV/r for 14 days (7 days vs. 12 days, *p* = 0.0010). In addition, the triple combination also alleviated symptoms completely within four days. The significantly better clinical and virological response was also reflected by the shorter median hospital stay in the combination group than in the control group (9 days vs. 14.5 days, *p* = 0.016).

A meta-analysis [38] recently concluded that treatment with LPV/r had no significant benefit in mortality and ARDS rates in COVID-19 patients, but, on subgroup analysis, the LPV/r group had a lower rate of ARDS, although this difference was not statistically significant (15.6% vs. 24.2%, *p* = 0.49). It is noteworthy that all included studies in this meta-analysis were observational, since the Cao’s RCT [33] was published after this search was conducted

### 3.6. Guideline Recommendations for LPV/r in COVID-19

Table 1 shows a synthesis of the guideline recommendations issued by the main scientific societies and agencies about LPV/r use in COVID-19.

### 3.7. Ongoing Interventional Trials for LPV/r in COVID-19

Table 2 shows a summary of the numerous ongoing interventional trials around the world investigating the efficacy of LPV/r in COVID-19 (available online at https://clinicaltrials.gov, accessed on 15 May 2020).

## 4. Discussion and Conclusions

To date, available evidence about the efficacy and the effectiveness of LPV/r in COVID-19 is poor and generally of low quality. There is only one RCT of adequate methodology conducted to address the topic whether adding LPV/r to the standard of therapy can bring clinical benefits [33], but, as previously discussed, the results should be read with great attention given the limited size of the enrolled sample; however, this RCT represents a research milestone for COVID-19, which demonstrates how in these dramatic pandemic times the scientific method can still be maintained. Another RCT recently published [37] has instead explored a different point of view, considering LPV/r as the standard of therapy (control group), and showed that the addition of ribavirin and interferon-β 1b to LPV/r confers clinical benefits.

Many scientific societies and agencies have used the GRADE approach and the consensus method to release their guidelines, and the recommendations have sometimes been expressed as “expert opinion”. Rightly, the results of the RCT of Cao et al. [33] are generally held in very high regard, despite several limits of this study, but our impression is that they “obscured” the data that emerged from several other studies, even if of lower quality, and from the very extensive clinical experience that has been made on the use of LPV/r from the beginning of the COVID-19 pandemic, with the efficacy of all alternative treatments still needing proofs.

Often the “net balance of recommendation” is uncertain, and LPV/r is recommended only in the context of a clinical trial. It should also be remembered that numerous ongoing interventional trials around the world are currently evaluating the efficacy of LPV/r in COVID-19.

In some cases, a recommendation clearly against the use has been expressed [39,40], in other cases it resulted more favorably [48,49,50,51]. In the past, the Public Health England has recommended LPV as a possible option for MERS patients, for its routinely availability and well-established favorable toxicity profile [19], even in the absence of strong evidence about the efficacy. Lacking to date effective treatment for COVID-19, and pending results of ongoing RCTs, in our opinion the same rational considerations of the favorable benefit/risk balance could also be prudently made for COVID-19.

We are fully aware of the seriousness of the current COVID-19 pandemic, and the lack of therapies of proven efficacy should in no way legitimize a failure of the scientific method. The validity of a given therapy must be properly demonstrated, but this applies both to its definitive approval and rejection. Consideration which are too hasty could have serious consequences.

In our opinion, at the current time, clinicians should not abandon the use of LPV/r for the treatment of COVID-19, pending results of numerous ongoing trials evaluating the efficacy of this drug.

## Figures and Tables

**Table 1:** Recommendations of the guidelines issued by international agencies and scientific societies about lopinavir/ritonavir use in COVID-19.

Guidelines, Year [Ref]	Recommendation	Strenght and/or Quality of Evidence	Grading of Recommendation	Net Balance of Recommendation
AMMI, CEPCPA, HPPC, 2020 [39]	We suggest not using LPV/r in patients with non-severe and severe COVID-19	Weak recommendation	GRADE approach	Against
SSC, 2020 [40]	In critically ill adults with COVID-19 we suggest against the routine use of LPV/r	Weak recommendation, low-quality evidence	GRADE approach	Against
SITA, SIP, 2020 [41]	At the present time, evidence from the first published RCT does not support off-label treatment with LPV/r in COVID-19 patients	One randomized trial	Consensus	Against/Uncertain
NIH, 2020 [42]	There are insufficient data to recommend either for or against the use of any antiviral or immunomodulatory therapy in patients with COVID-19 who have mild, moderate, severe, or critical illness (AIII). Except in the context of a clinical trial, the Panel recommends against the use of LPV/r because of unfavorable pharmacodynamics and negative clinical trial data (AI).	A: Strong recommendation I: One or more randomized trials III: Expert opinion	Consensus	Against/Uncertain
WHO, 2020 [43]	There is no current evidence to recommend any specific anti-COVID-19 treatment for patients with confirmed COVID-19. There are many ongoing clinical trials testing various potential antivirals.	Not indicated	Not indicated	Uncertain
DGIIN, DIVI, DGP, DGAI, 2020 [44]	We recommend to only use LPV/r (and other drugs) as part of compassionate use programs or approved study protocols after carefully evaluating risks and benefits for the individual patient	Not indicated	Consensus, multidisciplinary approach	Uncertain
ATS, 2020 [45]	For hospitalized patients with COVID-19 who have evidence of pneumonia, we make no suggestion either for or against treatment with LPV/r	30% for intervention, 26% no suggestion, 43% against intervention	CORE process	Uncertain
IDSA, 2020 [46]	Among patients who have been admitted to the hospital with COVID-19, the IDSA guideline panel recommends the combination of LPV/r only in the context of a clinical trial	Knowledge gap	GRADE approach	Uncertain
Australian Task force, 2020 [47]	For people with moderate to severe to critical COVID-19, only administer LPV/r in the context of clinical trials with appropriate ethical approval. Living Guidance	Evidence-based recommendation	Not indicated	Uncertain
CPAM, 2020 [48]	LPV/r orally, 2 capsules each time, twice a day, can be considered	Weak recommendation, low level of evidence	GRADE approach	Pro
KSID, KSAT, KSPID, 2020 [49]	Antiviral therapy may be considered for patients with confirmed COVID-19 and can be considered for patients with confirmed COVID-19 with moderate to severe course including pneumonia, those with worsening clinical findings, and those who are likely to progress to severe COVID-19 disease (elderly, chronic diseases, immunocompromised patients) (CIII). Antiviral agents should be administered as early as possible (BIII). LPV/r 400 mg/100 mg can be used up to twice a day, when used alone (CIII). Generally, antiviral agents are recommended for 7–10 days	B: Should generally be offered C: Optional III: Expert opinion or descriptive studies	GRADE approach	Pro
CCDCP, 2020 [50]	Antiviral treatment: LPV/r 200 mg/50 mg 2 pills twice a day, not exceeding 10 days	Not indicated	Not indicated	Pro
SIMIT, 2020 [51]	Recommended early start in case of: (1) age >70 years, and/or with comorbidities (2) mild-moderate symptoms with chest x-ray positive for pneumonia	Low-quality evidence	Consensus	Pro

Abbreviations: AMMI, Association of Medical Microbiology and Infectious Disease Canada; CEPCPA, Centre for Effective Practice and the Chinese Pharmaceutical Association; HPPC, Hospital Pharmacy Professional Committee; SSC, Surviving Sepsis Campaign; SITA, Italian Society of Anti-Infective Therapy; SIP, Italian Society of Pulmonology; NIH, National Institute of Health; WHO, World Health Organization; DGIIN, German Society of Medical Intensive Care and Emergency Medicine; DIVI, German Interdisciplinary Association of Critical Care and Emergency Medicine; DGP, German Respiratory Society; DGAI, German Society of Anaesthesiology and Intensive Care Medicine; ATS, America Thoracic Society; IDSA, Infectious Disease Society of America; CPAM, Evidence-Based Medicine Chapter of China International Exchange and Promotive Association for Medical and Health Care; KSID, Korean Society of Infectious Diseases: KSAT, Korean Society for Antimicrobial Therapy; KSPID, Korean Society of Pediatric Infectious Diseases; CCDCP, Chinese Centre for Disease Control and Prevention; SIMIT, Società Italiana di Malattie Infettive e Tropicali; GRADE, Grading of Recommendations, Assessment, Development and Evaluation; CORE, Convergence of Opinion on Recommendations and Evidence.

**Table 2:** Interventional trials investigating the efficacy of lopinavir/ritonavir in COVID-19.

Study Title	ClinicalTrials.gov Identifier	Interventions (LPV/r vs. or LPV/r Plus.)	Locations
Comparison of Lopinavir/Ritonavir or Hydroxychloroquine in Patients With Mild Coronavirus Disease (COVID-19)	NCT04307693	-Hydroxychloroquine	Korea
OUTpatient Treatment of COVID-19 in Patients with Risk Factor for Poor Outcome (OUTCOV)	NCT04365582	-Azithromycin -Hydroxychloroquine	France
Trial of Early Therapies During Non-Hospitalized Outpatient Window for COVID-19 (TREAT-NOW)	NCT04372628	-Hydroxychloroquine	USA
Treatments for COVID-19: Canadian Arm of the SOLIDARITY Trial (CATCO)	NCT04330690	-Remdesivir -Hydroxychloroquine	Canada
Clinical Trial to Evaluate Efficacy of Three Types of Treatment in Patients With Pneumonia by COVID-19 (Covid-19HUF)	NCT04346147	-Imanitib -Baricitinib -Hydroxychloroquine	Spain
Chemoprophylaxis of SARS-CoV-2 Infection (COVID-19) in Exposed Healthcare Workers (COVIDAXIS)	NCT04328285	-Placebo -Hydroxychloroquine	France
COVID MED Trial: Comparison of Therapeutics for Hospitalized Patients Infected With SARS-CoV-2 (COVIDMED)	NCT04328012	-Placebo -Hydroxychloroquine -Losartan	USA
Safety and Efficacy of Hydroxychloroquine + Favipiravir Drug Regimen in Comparison with Hydroxychloroquine + Kaletra on the Need for Intensive Care Unit Treatment in Patients with COVID-19	NCT04376814	-Favipiravir -Hydroxychloroquine	Iran
Effectiveness and Safety of Medical Treatment for SARS-CoV-2 (COVID-19) in Colombia	NCT04359095	-Azithromycin -Hydroxychloroquine -Standard treatment	Colombia
Efficacy and Safety of Umifenovir as an Adjuvant Therapy Compared to the Control Therapeutic Regiment of Interferon Beta 1a, Lopinavir/Ritonavir, and a Single Dose of Hydroxychloroquine in Moderate to Severe COVID-19: A Randomized, Double-Blind, Placebo-Controlled, Clinical Trial	NCT04350684	-Umifenovir -Interferon-β 1a -Hydroxychloroquine -Standards of Care	Iran
A Prospective/Retrospective, Randomized Controlled Clinical Study of Antiviral Therapy in the 2019-nCoV Pneumonia	NCT04255017	-Abidol hydrochloride -Oseltamivir	China
COVID-19 Ring-Based Prevention Trial with Lopinavir/Ritonavir (CORIPREV-LR)	NCT04321174	None	Canada
Efficacy of Pragmatic Same-day COVID-19 Ring Prophylaxis for Adult Individuals Exposed to SARS-CoV-2 in Switzerland (COPEP)	NCT04364022	-Hydroxychloroquine	Switzerland
Treatment of Moderate to Severe Coronavirus Disease (COVID-19) in Hospitalized Patients	NCT04321993	-Baricitinib -Hydroxychloroquine	Canada
Interferon Beta 1a in Hospitalized COVID-19 Patients (IB1aIC)	NCT04350671	-Interferon Beta-1A -Hydroxychloroquine	Iran
Evaluation of Efficacy of Levamisole and Formoterol+Budesonide in Treatment of COVID-19	NCT04331470	-Levamisole + Budesonide + Formoterol inhaler -Hydroxychloroquine	Iran
Evaluating and Comparing the Safety and Efficiency of ASC09/Ritonavir and Lopinavir/Ritonavir for Novel Coronavirus Infection	NCT04261907	-ASC09/ritonavir	China
Austrian CoronaVirus Adaptive Clinical Trial (COVID-19) (ACOVACT)	NCT04351724	-Hydroxychloroquine -Candesartan -Clazakizumab -Placebo -Other treatments	Austria
Antiviral Therapy and Baricitinib for the Treatment of Patients with Moderate or Severe COVID-19	NCT04373044	-Baricitinib -Hydroxychloroquine -Remdesivir	USA
Trial of Treatments for COVID-19 in Hospitalized Adults (DisCoVeRy)	NCT04315948	-Remdesivir -Interferon Beta-1A Hydroxychloroquine -Standard of care	France
Low Dose Anti-Inflammatory Radiotherapy for the Treatment of Pneumonia by COVID-19	NCT04380818	-Low-dose radiotherapy -Hydroxychloroquine -Tocilizumab -Azithromycin -Corticosteroid -LMWH	Spain
Lopinavir/Ritonavir, Ribavirin and IFN-beta Combination for nCoV Treatment	NCT04276688	-Ribavirin -Interferon Beta-1B	China
Various Combination of Protease Inhibitors, Oseltamivir, Favipiravir, and Hydroxychloroquine for Treatment of COVID-19: A Randomized Control Trial (THDMS-COVID-19)	NCT04303299	-Darunavir -Oseltamivir -Favipiravir -Hydroxychloroquine	Thailand
Randomised Evaluation of COVID-19 Therapy (RECOVERY)	NCT04381936	-Corticosteroid -Hydroxychloroquine -Azithromycin -Convalescent plasma -Tocilizumab	United Kingdom
